# An Epidemiological Analysis for Assessing and Evaluating COVID-19 Based on Data Analytics in Latin American Countries

**DOI:** 10.3390/biology12060887

**Published:** 2023-06-20

**Authors:** Víctor Leiva, Esdras Alcudia, Julia Montano, Cecilia Castro

**Affiliations:** 1 School of Industrial Engineering, Pontificia Universidad Católica de Valparaíso, Valparaíso 2362807, Chile; 2 Faculty of Statistics and Informatics, Universidad Veracruzana, Xalapa 91140, Mexico; esdras2304.ea@gmail.com (E.A.); julmontano@uv.mx (J.M.); 3 Centre of Mathematics, University of Minho, 4710-057 Braga, Portugal; cecilia@math.uminho.pt

**Keywords:** data science, epidemic models, reproduction number, SARS-CoV-2, time-series models

## Abstract

**Simple Summary:**

In this research, we investigate the COVID-19 spread in Latin American countries using time-series and epidemic models. We highlight the diverse outbreak patterns and the crucial role of the reproduction number in modeling pandemic scenarios. Our findings underscore the need for ongoing epidemic surveillance and accurate data handling.

**Abstract:**

This research provides a detailed analysis of the COVID-19 spread across 14 Latin American countries. Using time-series analysis and epidemic models, we identify diverse outbreak patterns, which seem not to be influenced by geographical location or country size, suggesting the influence of other determining factors. Our study uncovers significant discrepancies between the number recorded COVID-19 cases and the real epidemiological situation, emphasizing the crucial need for accurate data handling and continuous surveillance in managing epidemics. The absence of a clear correlation between the country size and the confirmed cases, as well as with the fatalities, further underscores the multifaceted influences on COVID-19 impact beyond population size. Despite the decreased real-time reproduction number indicating quarantine effectiveness in most countries, we note a resurgence in infection rates upon resumption of daily activities. These insights spotlight the challenge of balancing public health measures with economic and social activities. Our core findings provide novel insights, applicable to guiding epidemic control strategies and informing decision-making processes in combatting the pandemic.

## 1. Introduction

Severe acute respiratory syndrome coronavirus 2 (SARS-CoV-2), the virus responsible for the COVID-19 pandemic, was first identified in the city of Wuhan, China, in December 2019 [1,2]. Almost three and a half years after its detection, it can be said that this epidemic has plunged humanity into a state of confusion. The governments worldwide monitor the spread of the disease and take measures to contain and control the outbreaks without prior information. Therefore, it becomes crucial to assess and evaluate the best course of action to face this pandemic based on data analytics.

Every country has implemented measures to prevent the spread of COVID-19, with population isolation through quarantines gaining significant momentum. This study aims to assess the effectiveness of these measures in controlling the spread of the virus. In world history, this is the first time that a pandemic has compelled us into a complete state of global quarantine [3]. The coronavirus has infected over 750 million people and has caused the death of almost 7 million people as of the time of the present study [4]. A reliable and accurate dataset of the disease is crucial for scientists to conduct research and make informed decisions regarding policy development. Unfortunately, errors can occur in the data collection process, especially during a pandemic.

The coronavirus pandemic represents one of the most serious public health crises faced by the world, with Latin America being particularly hard-hit. The specific challenge lies in adapting existing protocols from previous epidemics to the unique characteristics of this virus. However, effectively controlling and managing outbreaks is vital due to the complexity involved. COVID-19 has had diverse impacts on both the globe and specifically in Latin America, extending beyond public health to affect various aspects, such as the economy of each country, as indicated by the gross domestic product, which represents the total output of goods and services within a nation [5]. The pandemic has left significant marks on the global level, but its economic and financial impact has been particularly profound in Latin America due to its unique historical backdrop.

In this study, we present a refined feedback process aimed at guiding governments in adopting effective health strategies to combat COVID-19. This process relies on robust data visualization sourced from multiple databases. Most countries have been tracking daily confirmed cases since the beginning, allowing for early reporting of disease incidence. The main challenge lies in ensuring an appropriate response while balancing political, health, and economic measures. Despite limited prior experience with COVID-19, certain crucial steps must be considered based on our accumulated knowledge and ongoing learning as the pandemic unfolds. Ongoing research offers the potential for a deeper understanding of virus’s behavior, transmission methods, and potential preventive measures, enabling the creation of predictive scenarios for similar situations in the future. This improved understanding can facilitate more informed decision-making, helping to mitigate the social impacts of the pandemic. After all, the global community was largely caught off guard by this pandemic, and measures used to curb its spread may have been implemented later than ideal. Previous research on similar events has demonstrated its value in decision-making for managing disease spread, contributing to more effective responses.

Time-series models, as explored and applied in [6,7,8], are particularly relevant in forecasting epidemic diseases, as evidenced in [9,10]. Studies into different aspects of epidemics is being conducted by numerous scientists, each striving to provide helpful insights that may benefit mankind. These studies employ a variety of methodologies, with some relying on epidemic models [11,12], while others predominantly use statistical and mathematical methods, as in [13], where the authors applied mathematical models to analyze and predict the timeline and phases of an epidemic, specifically focusing on COVID-19 in Italy. In addition, control theory [14] has been applied to epidemic models to derive optimal strategies for easing restrictive measures, as showcased in [15].

In addition to time-series and epidemic models, it is worthwhile to mention an alternative approach based on the geometric Brownian motion (GBM). In the context of disease spread modeling, the exponential growth of epidemic cases—as observed, for example, in the initial stages of spreading based on epidemic models—can be modeled with the GBM. Certain quarantine measures for such exponential-growth stochastic processes are then modeled as the so-called resetting, a partial reduction approach in the process magnitude at specifically chosen times (distributed, for instance, according to a Poisson model) [16,17,18]. Although this approach is not explored in the current study, it offers a valuable theoretical alternative that could provide additional insights into disease spread dynamics.

Nonpharmacological measures to combat COVID-19 have also been modeled in [19,20]. The same was performed in [21] using branching processes. Other research has focused on growth curves to analyze mortality and second waves of the pandemic [22,23]. Similarly, in [24], the generalized Richards and growth models were used to analyze the COVID-19 infected cases in China. In [25], a cluster analysis of COVID-19 mortality according to sociodemographic factors was carried out at municipal level in Mexico.

Machine learning models have been also employed, which are known for their flexibility and adaptability. Machine learning models have been successfully applied to complex phenomena, including cardiovascular diseases [26], and to make reliable predictions for emerging COVID-19 variants. For instance, as in [27], where the authors introduced a novel interpretable deep learning architecture to predict SARS-Cov-2 disease severity.

In the context of epidemics and pandemics, researchers have used machine learning models such as logistic regression, neural networks, and support vector machines to understand and predict the progression of COVID-19. A feature of these studies is their use of data from diverse regions around the world, highlighting the global reach of these epidemics [28,29,30]. Consequently, the goal of this study is to provide tools to bolster disease control efforts. As we approach the third year since the emergence of the pandemic, our primary objective is to enhance epidemiological surveillance across Latin America. We endeavor to comprehend the current status of the disease, evaluate the strategies deployed by different governments in response to this health crisis, and investigate the disease propagation in various Latin American nations that have implemented government-enforced quarantines. Furthermore, we aim to delineate the key parameters for managing the COVID-19 outbreak within these countries.

The threat level of COVID-19 is heavily dependent on the virus’s interaction with sensory receptors, meaning the myriad of coronavirus variants is determined by the interactions of the viral proteins with human receptors [31,32]. To date, COVID-19 has exhibited a variety of strains with diverse levels of contagion. Given the uncertainties surrounding the varying contagion levels of these strains, accurate identification of the virus lifecycle is vital for making informed health decisions, such as managing quarantines, designing effective vaccines [33], and setting appropriate national policies.

Considering the vulnerabilities of Latin American healthcare systems exposed by COVID-19, as demonstrated by the overburdening of healthcare systems and lack of access to treatments for a significant proportion of the population, the importance of thoroughly analyzing the virus’s propagation behavior and evaluating the implemented policies cannot be overstated. This critical evaluation is a prerequisite for devising the best strategies for disease containment. A notable approach [34] emphasized the need for efficient distribution of healthcare centers within each country based on parameters such as accessibility, demand, and equity. This optimization framework importantly should encompass the strategic allocation of vaccination centers to ensure the most effective response to the disease. Building upon these insights, our contributions include:

(i) Investigation of COVID-19 behavior in Latin America based on confirmed cases and deaths reported up until 31 December 2021.
(ii) Mapping of the incidence rate by country to assess COVID-19 in Latin America.
(iii) Forecasting of COVID-19 cases in Latin American countries until January 2022.
(iv) Comparison of the trend changes in COVID-19 by country, observing and describing the number of infection waves each country experienced.
(v) Formulation of the basic (instantaneous or effective) reproduction number ($R_{0}$) with values across different countries and the analysis of the effects of quarantine measures on transmission rates [35].
(vi) Proposal of an epidemic model to predict future disease spread, which can serve as a tool for developing predictive scenarios.

The rest of this article is organized as follows. Section 2 outlines the work strategy employed to accomplish the aforementioned objectives, including descriptions of each statistical method utilized. In Section 3, we detail the case study on our epidemiological analysis for assessing and evaluating COVID-19 in Latin America countries, showing the results obtained from implementing these methods. Finally, in Section 4, we offer a discussion and conclusions based on the findings, as well as suggestions for future research.

## 2. Methodology

In this section, we initially explain the process of estimating the instantaneous (or basic) reproduction number, followed by a detailed application of the susceptible, exposed, infectious, and recovered (SEIR) model [36]. Subsequently, we present the applied techniques based on statistical analysis of stochastic processes over time [37]. This includes a focused approach to time-series and trend estimation, with particular attention given to identifying shifts in these trends.

### 2.1. Estimation of the Instantaneous Reproduction Number

In [10], a methodology was proposed assuming that the infectious profile of a patient only depends on the time that has passed since the patient acquired the illness, rather than the time that has elapsed since the epidemic started. Given the instant *t*, we can represent the distribution of the number of infected people as

$$I\left(t\right)|\left(I\left(0\right),\cdots ,I\left(t-1\right),R\left(t\right),w\left(t\right)\right)∼\mathrm{Poisson}\left(\begin{array}{c} R\left(t\right)\sum_{s=1}^{t}I\left(t-s\right)w\left(s\right) \end{array}\right),$$

where w(t) is the probability distribution of the generation time of the outbreak, which can be considered as the probability distribution of the interval between successive cases of the illness [38]; and R(t) is the instantaneous reproduction number represented as

$$R\left(t\right)=\frac{E(I(t))}{\sum_{s=1}^{t}I\left(t-s\right)w\left(s\right)},$$

which is estimated by replacing the incidence expected by its observed value given by

$$\hat{R}\left(t\right)=\frac{I(t)}{\sum_{s=1}^{t}I\left(t-s\right)w\left(s\right)}.$$

The estimation of the reproduction number can be highly variable when the time interval is small at the time of interpretation. To address this issue, in [39], a process was proposed for parameter estimation using a Bayesian approach, assuming a specific probability distribution for the reproduction number. In this approach, a prior distribution is chosen to be a gamma probability model with a mean and standard deviation of 5, and a posterior distribution is obtained as

$$R\left(t\right)|\left(I\left(t-\tau +1\right),\cdots ,I\left(t\right),w\left(t\right)\right)\;∼\;\mathrm{Gamma}\left(1+\sum_{s=t-\tau +1}^{t}I\left(s\right),\;\frac{1}{5}+\sum_{s=t-\tau +1}^{t}\;\sum_{r=1}^{s}I\left(s-r\right)w\left(r\right)\right).$$

We use a window width of τ=7 days. The instantaneous reproduction number is estimated assuming a log-normal distribution with a mean of 4.7 days and a standard deviation of 2.9 days for the interval between successive cases. The implementation in the R software is provided by the function estimate_r from the EpiEstim package [10].

### 2.2. SEIR Model

One of the most critical challenges posed by the pandemic is the need to project the spread of the disease in the future and provide tools for better outbreak management in subsequent situations. In addition to the SEIR model, numerous other modeling methodologies have been utilized for COVID-19. For instance, susceptible, infectious, and recovered models, classical tools in epidemiology, have been refined and adapted for COVID-19 in several studies [40]. Machine learning models, capable of learning from complex, high-dimensional data, have been employed for forecasting the virus’s spread, using techniques such as regression, decision trees, and random forests [41]. Further, deep learning models, a subset of machine learning structures, have leveraged artificial neural networks to simulate the virus’s spread with a high degree of accuracy [42]. Each model has its unique strengths and limitations, and its applicability can depend on the specific objectives and constraints of a study. Given these considerations, we chose to utilize the SEIR model for our study and fit it as

$$\begin{array}{ccc} S^{\prime }\left(t\right) & = & -\beta S(t)I(t)/N\\ E^{\prime }\left(t\right) & = & \beta S(t)I(t)/N-\sigma E(t)\\ I^{\prime }\left(t\right) & = & \sigma E(t)-(\gamma +\mu )I(t)\\ R^{\prime }\left(t\right) & = & \gamma I(t), \end{array}$$

where $S^{\prime }\left(t\right)=dS\left(t\right)/dt$ and similarly for $E^{\prime }\left(t\right),I^{\prime }\left(t\right),R^{\prime }\left(t\right)$; β denotes the transmission rate of the disease; σ represents the rate at which individuals transit from being infected to being infectious; μ signifies the rate of mortality due to the disease; γ represents the recovery rate of individuals; and *N* represents the total population size. The SEIR model is adjusted using the transmission functions, denoted as

$$\beta_{t}=\left\{\begin{array}{cc} \beta , & \mathrm{if}t\leq t_{i};\\ \beta \left(\begin{array}{c} 1-\frac{\delta_{d}{\left(t-t_{i}\right)}^{2}}{1+\delta_{d}{\left(t-t_{i}\right)}^{2}} \end{array}\right), & \mathrm{if}t_{i}<t<t_{e};\\ \beta_{\min}+\left(\beta -\beta_{\min}\right)\left(\begin{array}{c} \frac{\delta_{i}{\left(t-t_{e}\right)}^{2}}{1+\delta_{i}{\left(t-t_{e}\right)}^{2}} \end{array}\right), & \mathrm{if}t>t_{e}; \end{array}\right.$$

where β is the initial transmission rate; *t* is the time from the first detected case; $t_{i}$ is the initial day of confinement minus a value equal to 2; $t_{e}$ is the end of confinement; $\delta_{d}$ is the rate of decrease of transmission; $\beta_{\min}$ is the value of $\beta_{t}$ at time $t_{e}$; and $\delta_{i}$ is the rate of increase of transmission after $t_{e}$.

If we consider the variation rate of the accumulated infected people as the rate at which individuals complete their exposed period, as suggested in [43], we can state that

$$C^{\prime }\left(T\right)=\sigma E\left(t\right),$$

where C(T) is an auxiliary variable that keeps track of the cumulative number of infectious individuals, and $C^{\prime }\left(T\right)$ keeps track of the curve of new cases (incidence). Therefore, the SEIR parameters are estimated by least squares [44] from the fit of the accumulated infected number and the number of accumulated confirmed cases until 31 December 2021, which is given as

$$\left(\hat{\beta },\hat{\mu },\hat{\sigma },\hat{\gamma },{\hat{\delta }}_{d},{\hat{\delta }}_{i}\right)=\mathrm{argmin}\sum_{i=1}^{T}{\left(C\left(t\right)-y_{t_{i}}\right)}^{2},$$

where $y_{t_{i}}$ is the time series that represents the observed number of accumulated confirmed cases at time $t_{i}$ and T=n days have elapsed since the first confirmed case [45].

### 2.3. Time-Series Models and Forecasting

ARIMA, standing for autoregressive integrated moving average, is employed in time-series analysis to comprehend and anticipate future trends [46,47,48]. In [49], it was argued that the ARIMA models must be suitable for dealing with complex and dynamic problems. These models use past observations for future predictions and incorporate unit root tests to check the stationarity of the series. The parameters of the ARIMA model are ascertained via the maximum likelihood (ML) estimation method, analogous to least squares, leading to efficient estimators. The ARIMA model is symbolized as ARIMA(p,d,q), where AR(*p*) signifies the autoregressive part of order *p*, I(*d*) is the degree *d* of differencing for stationarity, and MA(*q*) is the moving average part of order *q*. Then, respectively, *p*, *d*, and *q* represent the number of lagged observations, the level of differencing, and the lagged forecast errors. The ARIMA(p,d,q) model is defined as

$$\varphi \left(B\right)\left(1-B^{d}\right)Y_{t}=\varrho +\vartheta \left(B\right)\varepsilon_{t},$$

where φ(B) and ϑ(B) denote polynomials of orders p,q; $Y_{t}$ is a random variable with an observed value denoted by $y_{t}$; and $\varepsilon_{t}$ is the model random error. These polynomials should not have roots inside the unit circle, that is |B|<1, to ensure the model causality and invertibility. Note that ϱ is a constant, and a differencing polynomial of order *d* is included in the forecast when ϱ≠0 [6]. It is pertinent to note the backshift operator *B*, a notation used for lagged sequences. For a time series $y_{t}$, the lagged series is written as $By_{t}=y_{t-1}$, and in a broader sense, $B^{k}y_{t}=y_{t-k}$.

The ARIMA model assumes stationarity of the time series, implying a consistent mean and variance over time. The parameter *d* in the ARIMA model refers to differencing value, which is used to achieve stationarity if the series is initially non-stationary.

Differentiation converts the series into differences between consecutive observations $\left(y_{t}-y_{t-1}\right)$, and it can be applied more than once if the series remains non-stationary, as indicated by the parameter *d* in the ARIMA(p,d,q) model.

It is common to present the ARIMA(p,d,q) model in an alternate form as

$$Y_{t}=c+\phi_{1}y_{t-1}+\cdots +\phi_{p}y_{t-p}+\theta_{1}e_{t-1}+\cdots +\theta_{q}e_{t-q}+\varepsilon_{t},$$

where $Y_{t}$ is a random variable at time *t* and its observed values at t−1,⋯,t−p are $y_{t-1},\cdots ,y_{t-p}$, respectively; $\phi_{i}$ are the autoregressive parameters of the model; $\theta_{i}$ are the moving average parameters; and $\varepsilon_{t}$ is the model random error with observed values (residuals) at t−1,⋯,t−p being $e_{t-1},\cdots ,e_{t-p}$, respectively. The parameter *p*, the order of the autoregressive part, represents the number of lags of *Y* to be used as predictors. The parameter *d* is the order of integration, representing the number of times the data have had past values subtracted (also known as differencing), to make the time series stationary. Then, *q* is the order of the moving average part, representing the number of lagged forecast errors that should go into the ARIMA model.

The autocorrelation function (ACF) and partial autocorrelation function (PACF) are pivotal tools in time-series analysis, particularly for setting the parameters (p,q) of an ARIMA model. The ACF quantifies the correlation between time-series observations at different time points relative to the time lag between them, while the PACF determines the correlation between these observations when considering any correlations due to the values at shorter lags.

After identifying the model order (p,d,q), the parameters of the ARIMA model can be estimated, often via ML estimation. This estimation selects parameters that optimize the likelihood of the observed data given the model.

Suppose $y_{1},\cdots ,y_{n}$ is a time series of *n* observations. The likelihood function for an ARIMA model is defined as

$$L\left(\Theta ;y_{1},\cdots ,y_{n}\right)=f\left(y_{1},\cdots ,y_{n}|\Theta \right),$$

where Θ denotes the parameter vector to be estimated, and *f* is the joint probability density function of the observed data for the parameter Θ. Often, the log-likelihood function is used due to its mathematical ease, which is in our case given by

$$l\left(\Theta ;y_{1},\cdots ,y_{n}\right)=\log\left(L\left(\Theta ;y_{1},\cdots ,y_{n}\right)\right).$$

The likelihood function, *L* namely, quantifies how well a particular statistical model explains the observed data. In other words, it is a measure of how likely the observed data are, given the specific parameters of the model.

For an ARIMA model, the likelihood function assumes the errors at each time point follow a normal (or Gaussian) distribution. The likelihood function for the model parameters is calculated by evaluating the normal probability density function at each data point and multiplying these densities for the errors because they are a white noise (independent). Mathematically, for a sample of size *n* and errors (independent) $\varepsilon_{1},\cdots ,\varepsilon_{n}$, the likelihood function is stated as

$$L\left(\phi ,\theta ,\varsigma^{2}\right)=\prod_{i=1}^{n}\frac{1}{\sqrt{2\pi }\varsigma }\exp\left(-\frac{\varepsilon_{i}^{2}}{2\varsigma^{2}}\right).$$

The ML estimates are the parameter values that maximize this log-likelihood function. The parameters to be estimated in an ARMA(p,q) model include autoregressive coefficients $\phi_{j}$, for j∈{1,⋯,p}, moving average coefficients $\theta_{j}$, for j∈{1,⋯,q}, and the error term variance $\varsigma^{2}$.

Different approaches to the initial values of the estimation process, which are usually unknown, can result in different likelihood versions and parameter estimates. Due to the high dimensionality of the parameter space and potential multiple local maxima, numerical optimization methods are generally used to find the ML estimates.

For ARIMA models, automatic model selection can optimize predictive accuracy. The model order (p,d,q) can be selected minimizing the values of the Akaike (AIC) or Bayesian (BIC) information criteria expressed as

$$\mathrm{AIC}=-2\log\left(L\right)+2\left(p+q+k+1\right),\;\mathrm{BIC}=\mathrm{AIC}+\left(\log(n)-2\right)\left(p+q+k+1\right),$$

where k=1, if μ≠0, and k=0, if μ=0.

Through an automatic ARIMA modeling process, beneficially implemented in R [50], models were selected based on precision, using criteria such as mean absolute percentage error (MAPE), mean absolute deviation (MAD, also known as mean absolute error), and mean squared deviation (MSD, also known as mean squared error) to distinguish the best forecasts. These are common metrics used in statistics and machine learning to measure the accuracy of predictions or forecasts, especially in the context of regression and time-series analysis. This automatic process, outlined in Algorithm 1, enables an efficient and systematic approach to data modeling. The baseline model, representing future COVID-19 case forecasts, is presented as in [51].
**Algorithm 1** Automatic ARIMA modeling procedureStep 1:Select *d*, for 0≤d≤2, using the Kwiatkowski–Phillips–Schmidt–Shin unit root test.Step 2:Obtain *p*, *d*, and ϱ by minimizing the AIC after differencing the data *d* times.Step 3:Apply a stepwise search through model space to generate a simple model.

In this work, we apply ARIMA models to forecast time series of COVID-19 cases in a representative sample of Latin American countries, that is, Argentina, Bolivia, Brazil, Chile, Colombia, Ecuador, Paraguay, Peru, and Uruguay, in South America; Dominican Republic in the Caribbean; Costa Rica and Belize in Central America; and Mexico in North America. This selection of countries was driven by the availability of COVID-19 data.

The time series is broken down into its components (data, trend, seasonality, and remainder) for visualization purposes and was used to predict COVID-19 cases for January 2022. In the process, the Dickey–Fuller and Ljung–Box tests [52] were employed to evaluate stationarity and examine autocorrelation, respectively [53]. Following these tests, we generated the ACF and PACF to initiate the order determination for the ARIMA models.

Another prevalent method for forecasting time series employed in this study is the Holt-Winters method [54]. Renowned for its efficacy with time-series data exhibiting trends and seasonal patterns, it serves as a valuable tool in our analysis. We used this approach as a comparative measure, scrutinizing its predictive abilities against those of the ARIMA models. The Holt-Winters method is characterized by three parameters: level (α), trend (ζ), and seasonality (λ). The model consists of two variations: additive and multiplicative. On the one hand, in the additive model, the forecast equation at t+h is represented as

$${\hat{y}}_{t+h}=l_{t}+hb_{t}+s_{t-m}+h_{m}^{+},$$

where $l_{t}$ is the level, $b_{t}$ is the trend, and $s_{t}$ is the seasonal component. Note that *h* is the forecast horizon; *m* represents the seasonal length; and $h_{m}^{+}$ is the smallest integer greater than or equal to h/m.

The level, trend, and seasonal equations at *t* are formulated as

$$\begin{array}{ccc} l_{t} & = & \alpha \left(y_{t}-s_{t-m}\right)+\left(1-\alpha \right)\left(l_{t-1}+b_{t-1}\right)\\ b_{t} & = & \beta \left(l_{t}-l_{t-1}\right)+\left(1-\zeta \right)b_{t-1}\\ s_{t} & = & \lambda \left(y_{t}-l_{t}\right)+\left(1-\lambda \right)s_{t-m}. \end{array}$$

The multiplicative model, on the other hand, uses the forecast equation at t+h established as

$${\hat{y}}_{t+h}=\left(l_{t}+hb_{t}\right)s_{t-m}+h_{m}^{+}.$$

The level, trend, and seasonal equations at *t* are slightly different and presented as

$$\begin{array}{ccc} l_{t} & = & \alpha \left(\frac{y_{t}}{s_{t-m}}\right)+\left(1-\alpha \right)\left(l_{t-1}+b_{t-1}\right)\\ b_{t} & = & \zeta \left(l_{t}-l_{t-1}\right)+\left(1-\zeta \right)b_{t-1}\\ s_{t} & = & \lambda \left(\frac{y_{t}}{l_{t}}\right)+\left(1-\lambda \right)s_{t-m}. \end{array}$$

In all these equations, α, ζ, and λ are constants that must be optimized.

### 2.4. Trend Estimation

To estimate the daily trend of COVID-19 cases in Latin America, we utilize a time-series model [9] expressed as

$$\hat{m}=\frac{1}{\left(2d+1\right)}\sum_{i=-d}^{d}y_{t+i},$$

where $y_{t}$ represents the confirmed COVID-19 cases at time *t*, and the estimated trend, $\hat{m}$ say, is determined by a moving average [9,10]. The constant *d* governs the range and degree of smoothing [9]. Decreasing *d* results in smoother moving averages that capture trends effectively but may induce false alarms. Conversely, increasing *d* minimizes false alarms but may compromise the identification of ongoing trends [9,51]. Given the nature of our data, we set d=7, which corresponds to a 15-day window for trend estimation.

### 2.5. Detection of Trend Shifts

In epidemiological studies, the utilization of statistical models is crucial for understanding the direct impacts of diseases on various social dimensions, including global markets [5]. A valuable tool in these endeavors is the breakpoint methodology, used for estimating shifts in trends. In the context of the COVID-19 pandemic, we adopted this approach to discern trend changes across Latin American countries reporting confirmed cases.

Given the unique disease progression in each country, modulated by factors such as initial exposure date and implemented containment measures, we were able to identify shifts in trends regarding confirmed cases and fatalities.

Following [10], the detection of trend periods is simplified by identifying the breakpoints that divide the series. For a time series, $y_{t}$ namely, with a linear trend, we hypothesize multiple segments, each potentially displaying a distinct trend. This is represented using a piecewise linear regression model stated as

$$Y_{t}=\left\{\begin{array}{cc} \alpha_{1}+\zeta_{1}t+\epsilon_{t}, & t\in \{1,\cdots ,p\};\\ \alpha_{2}+\zeta_{2}t+\epsilon_{t}, & t\in \{p+1,\cdots ,T\}. \end{array}\right.$$

In this formulation, $\alpha_{1},\alpha_{2},\zeta_{1},\zeta_{2}$ are the regression model coefficients, and ϵ(t) represents a random perturbation with zero mean and constant variance.

To establish *p* as a change point, we test the hypotheses presented as

$$\begin{array}{c} H_{0}:\alpha_{1}=\alpha_{2},\;\zeta_{1}=\zeta_{2}\\ H_{1}:\alpha_{1}\neq \alpha_{2},\;\zeta_{1}\neq \zeta_{2}. \end{array}$$

The corresponding F-statistic enables us to contrast the residuals of the piecewise model against an unsegmented model given by

$$Y_{t}=\alpha +\zeta t+\epsilon_{t}.$$

As pointed out in [55,56], we might not know the exact number of breakpoints in a real-world time series. Therefore, a test is proposed to verify if the series has only *m* breakpoints, leading to a piecewise linear model formulated as

$$Y_{t}=\alpha_{j}+\zeta_{j}t+\epsilon_{t},\;t=t_{j-1}+1,\cdots ,t_{ji},\;j\in \left\{1,\cdots ,m+1\right\},$$

where $t_{0}=0$ and $t_{m+1}=n$ [56].

Denote the *m* breakpoints identified in the test as $i_{1},\cdots ,i_{m}$. Then, we define the sum of squared residuals (SRS) for the segments of the model as

$$\mathrm{SRS}\left(i_{1},\cdots ,i_{m}\right)=\sum_{j=1}^{m+1}\mathrm{SRS}\left(i_{j-1},i_{j}\right),$$

where $\mathrm{SRS}(i_{j-1},i_{j})$ represents the SRS for a given segment, between $i_{j-1}$th and $i_{j}$th breakpoints. This SRS is calculated by fitting the segmented model to that specific interval and summing the squares of the differences between the observed values and the values predicted by the model. As a result, we have that

$$\left({\hat{i}}_{1},\cdots ,{\hat{i}}_{m}\right)=\mathrm{argmin}\;\mathrm{SRS}\left(i_{1},\cdots ,i_{m}\right).$$

This methodology is applied as a complementary element of the trend estimation, enhancing our understanding of the time-series trend shifts. We used the function breakpoints from the strucchange package of the R statistical software for this purpose [50,57]. The identified breakpoints are visually represented in the time-series plot using blue and red panels, where each panel denotes a period with the trend remaining consistent.

## 3. Case Study

In this section, we present the main results obtained based on the methodology outlined in Section 2 and the analyses conducted throughout that section. We conduct an exploratory phase of our analysis, which involved the creation of choropleth maps to represent the distribution of COVID-19 cases across the Latin American countries upon study. To estimate non-stationarity, we used a moving average model, enabling us to identify significant shifts in the time-series trends. Subsequently, we calculated the real-time reproduction number using a Bayesian method. This number was superimposed on the quarantine timelines for each country in our study to assess the behavior of $R_{0}$ during and post confinement. To conclude our analysis, we deploy a SEIR model to determine the epidemiological curves based on realistic estimates of exposed and infected individuals.

### 3.1. Data, Methodology, and Software

The data used for this analysis comprise cases and fatalities confirmed by COVID-19 on a national level in Latin America, drawn from public data repositories at John Hopkins University and Our World in Data. The open-source repositories can be secured from https://github.com/CSSEGISandData/COVID-19 (accessed on 20 May 2023), https://github.com/owid/covid-19-data (accessed on 15 April 2022).

We have also consulted the institutional websites of each country to gather officially recognized dates for the implementation of quarantines. The dataset encompasses the period from 23 February 2020, when the first COVID-19 case was confirmed among the thirteen countries included in this study, to 31 December 2021.

**Figure 1:**
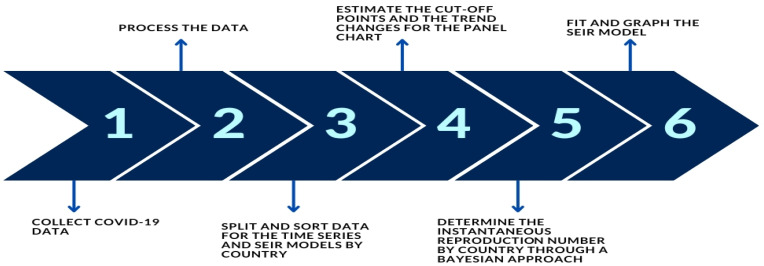
Proposed methodology. Source: The authors.

Our methodology is summarized in Figure 1.

We utilize the Tableau software [58] for the development of the exploratory analysis graphs, and the R software [50] was instrumental in producing data summaries and executing the aforementioned methodologies. Data handling was performed using the dplyr, openxlsx, reshape2, xtable, and tidyverse packages. Forecasting of time series incorporated the forecast, tseries, TTR, lubridate, and zoo packages, while the ggplot2 package was utilized for the design of graphs.

### 3.2. Exploratory Data Analysis

**Figure 2:**
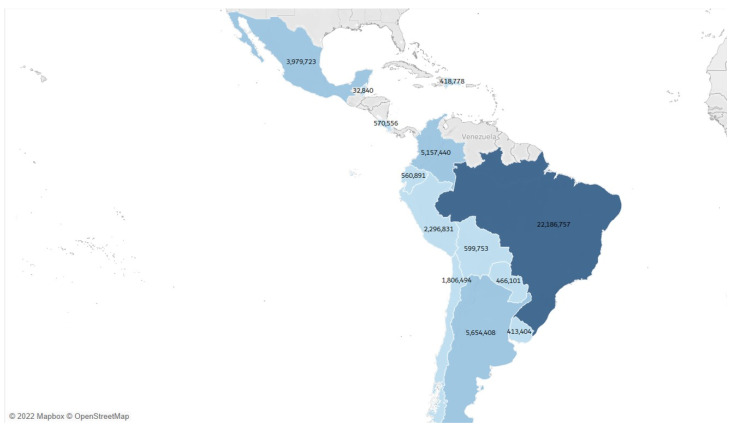
Latin America choropleth map of COVID-19 accumulative confirmed cases. The colors show a scale of confirmed cases, being the color with the lowest tonality the country with the fewest cases, until 31 December 2021. Source: The authors produced with 2022 Mapbox OpenStreetMap.

The results presented next highlight the distribution of confirmed COVID-19 cases across Latin America. Figure 2 illustrates that Brazil had the highest number of confirmed cases throughout the first two years of the pandemic. It is notable that neighboring countries generally exhibited lower infection rates compared to Brazil, with the exception of Colombia, which experienced a significant number of cases. Moreover, Belize, with its smaller population size, had the lowest number of cases, as evident when considering the incidence rate.

**Figure 3:**
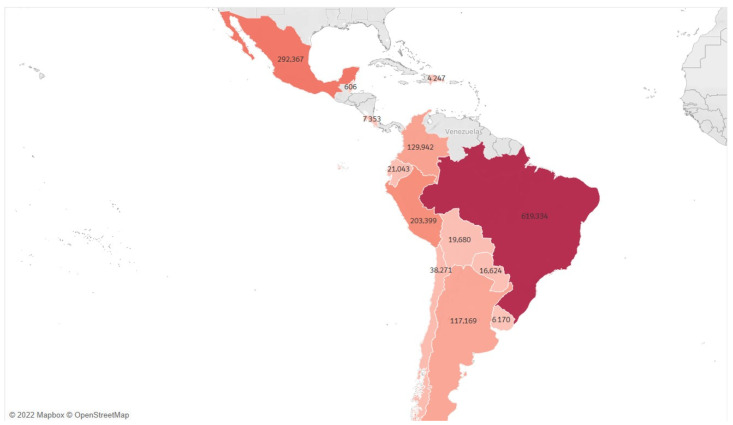
Latin America choropleth map of COVID-19 accumulative deaths. The colors show a scale of confirmed deaths, being the color with the lowest tonality the country with the fewest cases, until 31 December 2021. Source: the authors produced with 2022 Mapbox OpenStreetMap.

In Latin America, the deaths have been high [59], particularly in Brazil [9] and Chile [10]. However, there is no consistent relationship between the number of deaths and the number of confirmed cases in each country, except for Brazil and Belize, which have the highest and lowest numbers of confirmed cases and deaths, respectively (Figure 3). For instance, Peru and Chile have similar numbers of confirmed cases (2,296,831 and 1,806,494, respectively), but Peru has reported 203,399 confirmed deaths compared to Chile that reported 38,271. Similarly, Costa Rica and Ecuador both have around 570,556 confirmed cases, but Costa Rica reported 7353 deaths while Ecuador informed 21,043.

To accurately assess the situation in each country, it is important to consider the national incidence rate, which takes into account the population size. It is crucial to recognize that having 30,000 cases in a population of 300,000 is different from having 30,000 cases in a population of 3,000,000. Failing to calculate the incidence rate can lead to incorrect conclusions. Therefore, the incidence rate is calculated by adjusting the number of confirmed cases and deaths based on the population size in each country.

**Figure 4:**
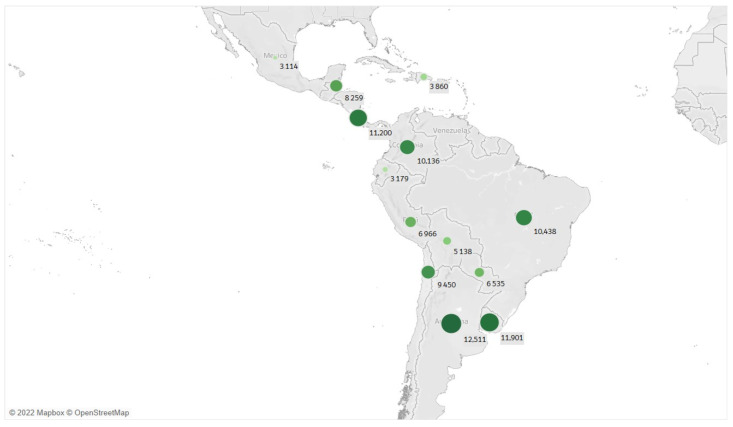
Latin America dot map of COVID-19 incidence rate by country. The size of every dot shows the incidence rate level, being the dot with the highest size the country with the highest incidence rate, until 31 December 2021. Source: the authors produced with 2022 Mapbox OpenStreetMap.

When examining the incidence rate adjusted by the population size, it reveals interesting insights into the impact of COVID-19 across different countries. Surprisingly, countries such as Argentina, Uruguay, Costa Rica, and Colombia show higher incidence rates compared to Brazil, despite not having the highest number of cases. In addition, Mexico, Colombia, and Ecuador have lower incidence rates, indicating a more consistent relationship between confirmed cases and population size in these countries (Figure 4).

### 3.3. Epidemic Model

**Figure 5:**
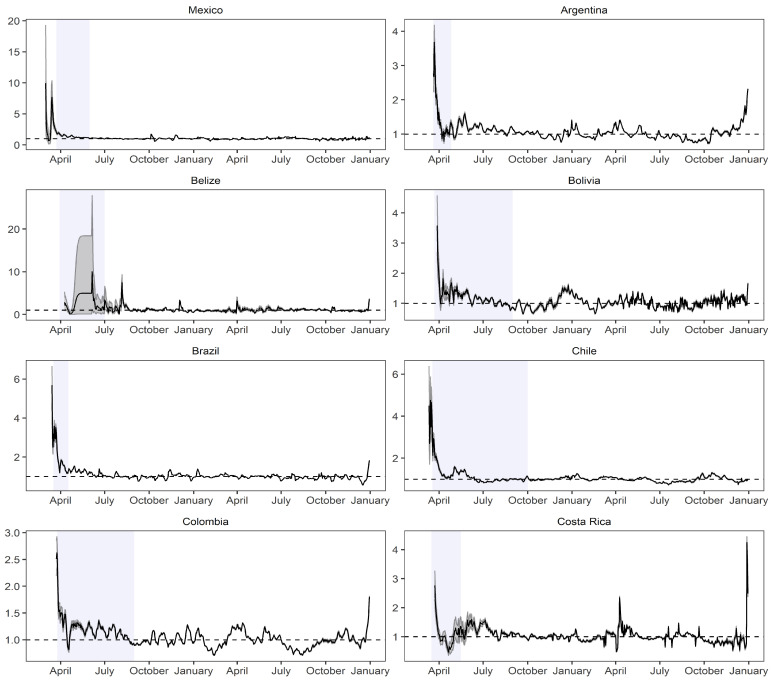
Instantaneous reproduction number estimated implementing its posterior median and disaggregated by country ranging from Mexico to Costa Rica. Source: The authors.

**Figure 6:**
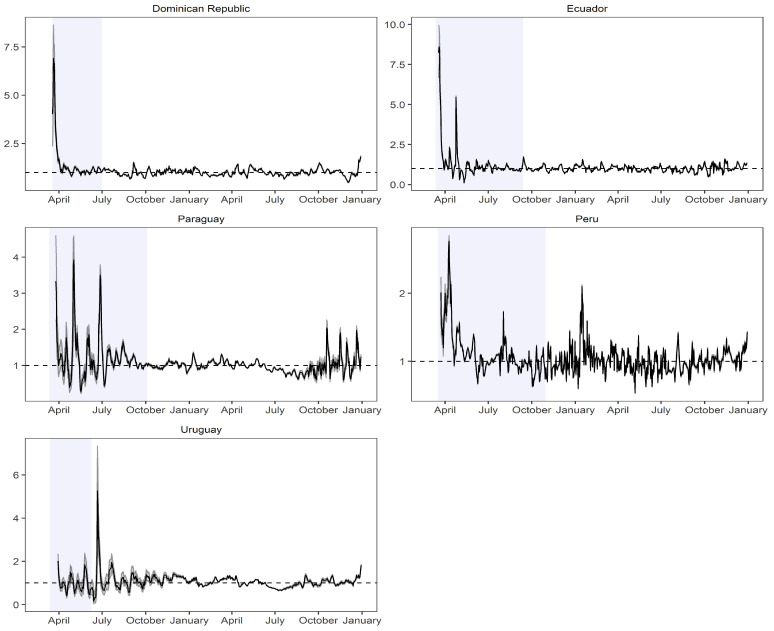
Instantaneous reproduction number estimated implementing its posterior median and disaggregated by country ranging from Dominican Republic to Uruguay. Source: The authors.

Figure 5 and Figure 6 illustrate the behavior of the instantaneous reproduction number ($R_{0}$) by presenting its posterior median along with the corresponding posterior distribution of the parameter. The blue panels superimposed on the figures represent the periods of quarantine, allowing us to observe the impact of these measures on $R_{0}$ during and after the implemented confinement.

In countries such as Argentina, Belize, Bolivia, Brazil, Chile, Dominican Republic, and Ecuador, the implementation of quarantine measures led to a significant decrease in the instantaneous reproduction number. However, in Peru and Uruguay, despite having similar quarantine periods (and longer in some cases), $R_{0}$ did not decrease significantly.

After the end of the quarantine period, Uruguay experienced a notable increase in $R_{0}$, while Peru exhibited a varying behavior with frequent fluctuations throughout the studied period. The highest values of $R_{0}$ were observed in Mexico and Belize, ranging from 10 to 20 units, indicating that one infected individual could potentially transmit the virus to a range between 10 and 20 other people. In addition, Peru, Paraguay, Costa Rica, Bolivia, and Argentina had lower reproduction values, with a maximum value of 4 units, implying lower transmission rates. For the SEIR model, Colombia was considered due to its relatively small population size compared to other countries such as Mexico and its high level of contagion. An initial confinement period was defined from the 19th day (counting from the first confirmed case) and lasted until the 178th day.

**Figure 7:**
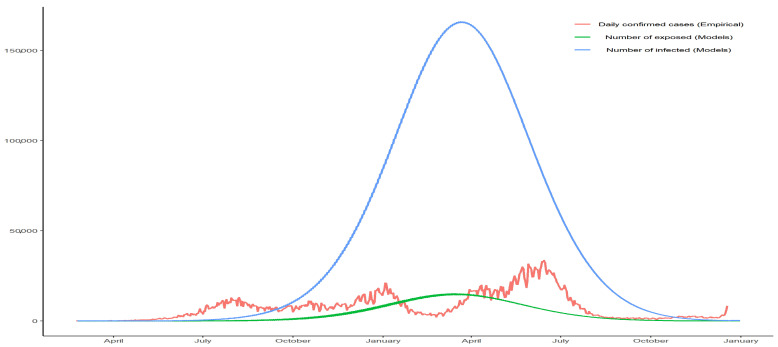
Prevalence of infected cases in Colombia and its relationship with the daily confirmed cases. Source: the authors.

The SEIR model parameters were fitted based on the criteria earlier described using data up to 31 December 2021. Figure 7 shows the prevalence of COVID-19 infected cases in Colombia and its relationship with the daily confirmed cases. The estimates of the SEIR parameters are: $\hat{\beta }=0.3250$, $\hat{\mu }=0.0002$, $\hat{\sigma }=1.3608$, $\hat{\gamma }=0.2363$, ${\hat{\delta }}_{d}=0.0000$, and ${\hat{\delta }}_{i}=0.0603$. The fitting and estimation of the SEIR model provide valuable insights into the prevalence of the disease, capturing the true number of infections at a given time.

The model estimates a significant difference between the confirmed cases reported and the COVID-19 number of infections. This disparity is evident, with the model estimating up to 60,000 infected individuals compared to the recorded peak of nearly 30,000 cases during the epidemic. These findings highlight the importance of accounting for undetected or unreported cases and emphasize the need for comprehensive testing and surveillance measures to accurately assess the true extent of the epidemic.

### 3.4. Main Results

Next, we present the main results obtained based on the methodology outlined in Section 2 and the analyses conducted throughout that section.

For forecasting purposes, Uruguay was chosen as an example. ARIMA models with various orders were fitted, and the AIC was used to select the most appropriate model. The model with the lowest AIC value was chosen. The accuracy of the models was evaluated using the MAPE and the ARIMA (3,1,1) structure.

To determine the best forecast model, an automatic ARIMA model was also fitted and compared to the previously selected model. Three models were evaluated for forecasting: Holt-Winters, automatic ARIMA, and ARIMA (3,1,1). After conducting evaluations using the same parameters, Holt-Winters was identified as the best-fitted model and provided the most accurate forecast. Consequently, the forecast was generated using the Holt-Winters model up until January 2022.

**Figure 8:**
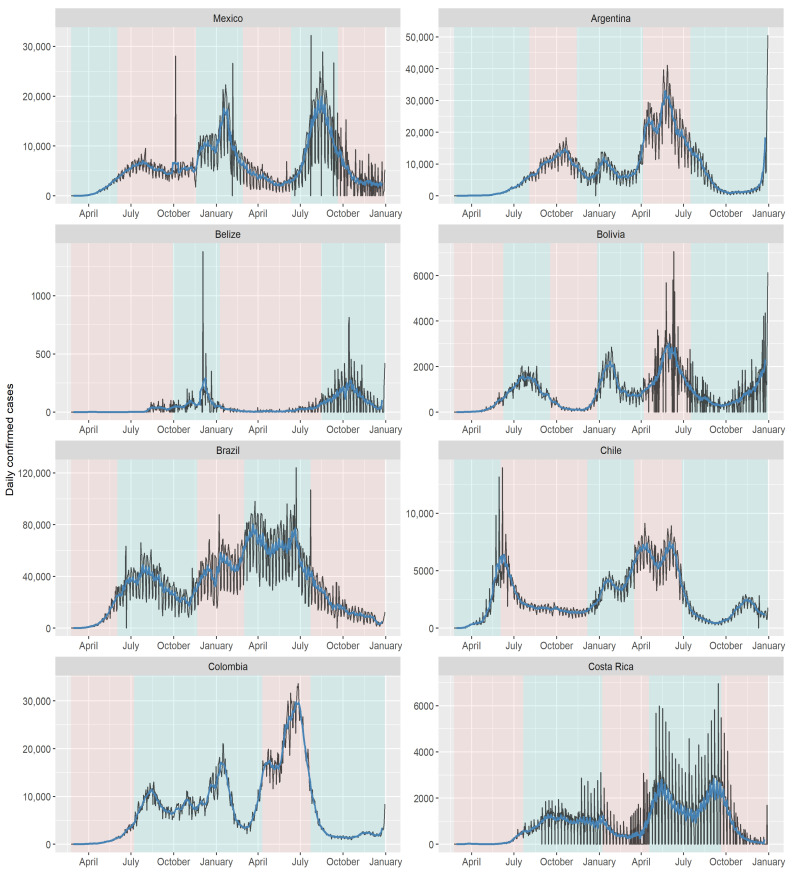
COVID-19 cases from Mexico to Costa Rica with the moving average superimposed and the cut-off points from Table 1. The vertical axis of each graph has an own scale according the country. Source: the authors.

**Figure 9:**
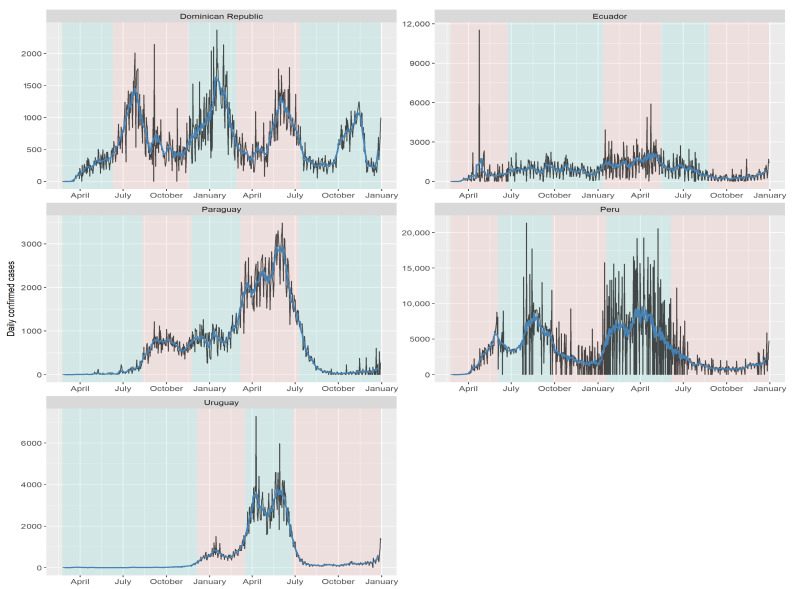
COVID-19 cases from Dominican Republic to Uruguay with the moving average superimposed and the cut-off points from Table 1. The vertical axis of each graph has an own scale according the country. Source: the authors.

By comparing the forecast made with the model to the behavior of the disease in the first month of 2022, we can observe a remarkably accurate fit to the empirical data. The forecasted values closely align with the observed trends and patterns, validating the effectiveness of the chosen model in capturing the dynamics of the disease. Note that in countries where the first wave occurred between June and July, there was a noticeable pattern of sharp increases and decreases in COVID-19 cases throughout 2020 and 2021. This can be observed in the distinct trends displayed by each country in Figure 8 and Figure 9, with Dominican Republic exhibiting one of the most significant trend changes. Ecuador and Belize experienced relatively stable case numbers without a prominent surge or decline. Furthermore, we identify plateau periods in all countries, indicating the impact of implemented quarantines by the respective health authorities. These stability periods can be compared to the estimated instantaneous reproduction number superimposed on the quarantine periods [60], revealing the effectiveness of quarantine measures in controlling the spread of the disease. It is worth highlighting the situation in Uruguay, where the onset of COVID-19 waves occurred later compared to other countries. Despite experiencing a significant wave when the disease initially entered the country, Uruguay generally maintained a low infection in subsequent periods.

The present analysis of each Latin American country clearly demonstrates heterogeneity, as evident from the identified cutoff dates presented in Table 1. Notably, there is similarity in the trend changes among Mexico, Brazil, Chile, and Dominican Republic, as observed in Figure 8 and Figure 9. Table 1 reveals that the first wave of COVID-19 cases occurred in Mexico, Bolivia, Brazil, Chile, Colombia, and Peru in the early days of June. Similarly, Colombia, Costa Rica, and Ecuador experienced their first trend change in late June and early July. Argentina, Belize, and Paraguay encountered their first wave in late August and early September. Uruguay had the latest onset of the COVID-19 wave, with cases emerging in early December. The impact of implemented quarantines on the population is reflected in the dynamics of the instantaneous reproduction number [56].

## 4. Discussion and Conclusions

The central objective of this article was to scrutinize the spread of COVID-19 across Latin America and assess the effectiveness of implemented quarantines. Through comprehensive time-series analysis and the evaluation of trend alterations via moving averages, we uncovered distinct patterns in COVID-19 cases across neighboring countries. A compelling example of this emerged in the comparison of Mexico and Belize, which exhibited significant differences in their outbreak spread behaviors. These disparities could be attributed to variations in health infrastructure and economic strength, with Mexico typically having more robust systems in place. Similarly, our examination revealed parallel trend patterns between Colombia and Peru, as well as with Peru and Bolivia. Such patterns could be influenced by unique government strategies and policies enacted in these countries, highlighting the significance of diverse factors such as the timing and stringency of measures when assessing COVID-19 dynamics.

A unique contribution of our research lies in the methodological approach we adopted, utilizing time-series analysis and moving averages. This provided a refined understanding of the pandemic’s trajectory and enabled us to dissect differences in outbreak spread behaviors among neighboring countries. It also allowed us to study the impact of various factors, including population size and public health policies, on these behaviors.

In assessing the data, it was evident that larger nations, such as Brazil and Mexico, experienced a high burden of infections. Yet, smaller countries, such as Colombia, outpaced Mexico in confirmed cases. This discrepancy underscores the fact that factors beyond population size can influence the spread and severity of the disease. Furthermore, the absence of a directly proportional relationship between the number of cases and deaths in some nations warrants further exploration. This observation underscores the necessity for in-depth research and more comprehensive studies like our current work, to understand the complex dynamics of COVID-19 spread and the multitude of influencing factors.

**Figure 10:**
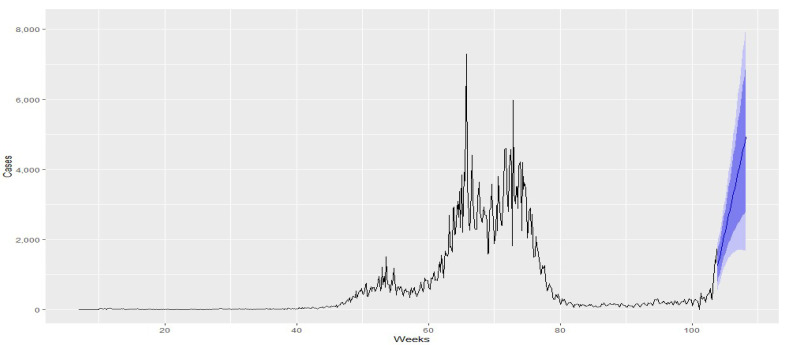
Forecast through the Holt-Winters model in Uruguay to January 2022. Source: the authors.

**Figure 11:**
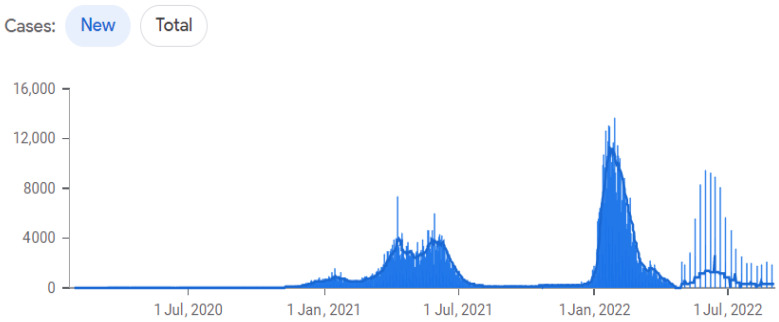
COVID-19 cases in Uruguay until 20 July 2022. Source: John Hopkins University.

Our research underscores the effectiveness of the forecast models used, as demonstrated in the comparison presented in Figure 10 and Figure 11. These models have proven to be valuable tools in understanding the disease’s behavior and forecasting its future trajectory. The novelty of our contribution lies in applying these models specifically within the Latin American context, providing a deeper understanding of the region’s situation.

The study draws parallels with the observation given in [39] regarding the pivotal role of the reproduction number in epidemic control strategies. Despite a decrease in this number following quarantine measures, we found a consistent increase in confirmed COVID-19 cases, indicating the disease’s spread was not effectively halted. This was particularly notable in Uruguay, where a more extended quarantine period could potentially have suppressed both the infection surge and reproduction number. These findings suggest concrete recommendations for policy-makers and public health officials, underlining the importance of a careful and gradual resumption of everyday activities after the quarantine.

This article considered the COVID-19 incidence by accounting for new cases in Latin America and considering the incidence rate and population size per country. The primary focus was to analyze and compare the spread of the disease between countries. However, we recommend a comprehensive analysis that incorporates the incidence rate for more accurately evaluating COVID-19 severity in future research.

By modifying the SEIR model to incorporate time-dependent transmission rates, we provided a closer approximation of the epidemiological situation. In Colombia, for instance, the estimated infection curve was approximately double the number of registered cases, revealing a critical discrepancy that needs to be addressed for improved epidemiological surveillance.

Throughout this work, the different realities experienced during the pandemic in Latin America were demonstrated. This work reaffirms the critical role of statistical and mathematical methodologies in understanding and addressing outbreaks. Yet, as new data and insights become available, continuous refinement and updating of these methodologies is required.

Future research should consider alternative methodological approaches not explored in this article, such as principal component analysis for country classification [12,59,61], as well as a theoretical approach based on the geometric Brownian motion [16,17,18]. Such studies can foster the development of more comprehensive, effective, and region-specific response strategies to future outbreaks.

## Figures and Tables

**Table 1:** COVID-19 confirmed cases cut-off points by country. Source: the authors.

Country	Cut-Off Point				
	1st	2nd	3rd	4th	5th
Argentina	3 August 2020	13 November 2020	5 April 2021	16 September 2021	N/A
Belize	29 September 2020	9 November 2020	15 August 2021	N/A	N/A
Bolivia	8 June 2020	17 September 2020	27 December 2020	7 April 2021	17 June 2021
Brazil	2 June 2020	21 November 2020	2 March 2021	23 September 2021	N/A
Chile	2 June 2020	6 December 2020	17 March 2021	28 June 2021	N/A
Colombia	8 July 2020	10 April 2021	23 June 2021	N/A	N/A
Costa Rica	21 June 2020	7 January 2021	18 April 2021	20 September 2021	N/A
Dominican Republic	10 June 2020	18 November 2020	27 February 2021	12 July 2021	N/A
Ecuador	24 June 2020	12 January 2021	15 May 2021	24 August 2021	N/A
Mexico	2 June 2020	18 November 2020	27 February 2021	11 June 2021	20 September 2021
Paraguay	11 August 2020	23 November 2020	7 March 2021	9 July 2021	N/A
Peru	3 June 2020	25 September 2020	19 January 2021	5 June 2021	N/A
Uruguay	6 December 2020	17 March 2021	26 June 2021	N/A	N/A

## Data Availability

Not applicable.
